# Emerging Therapy for Diabetic Cardiomyopathy: From Molecular Mechanism to Clinical Practice

**DOI:** 10.3390/biomedicines11030662

**Published:** 2023-02-22

**Authors:** Chin-Feng Hsuan, Sean I. F. Teng, Chih-Neng Hsu, Daniel Liao, Allen Jiun-Wei Chang, Hsiao-Lin Lee, Siow-Wey Hee, Yi-Cheng Chang, Lee-Ming Chuang

**Affiliations:** 1 Division of Cardiology, Department of Internal Medicine, E-Da Hospital, I-Shou University, Kaohsiung 824, Taiwan; 2 Division of Cardiology, Department of Internal Medicine, E-Da Dachang Hospital, I-Shou University, Kaohsiung 807, Taiwan; 3 School of Medicine, College of Medicine, I-Shou University, Kaohsiung 824, Taiwan; 4 Department of Cardiology, Ming-Sheng General Hospital, Taoyuan 330, Taiwan; 5 Division of Cardiology, Department of Internal Medicine, National Taiwan University Hospital Yunlin Branch, Yunlin 640, Taiwan; 6 Graduate Institute of Medical Genomics and Proteomics, College of Medicine, National Taiwan University, Taipei 100, Taiwan; 7 Department of Internal Medicine, Division of Endocrinology and Metabolism, National Taiwan University Hospital, Taipei 100, Taiwan; 8 Institute of Biomedical Sciences, Academia Sinica, Taipei 115, Taiwan; 9 Graduate Institute of Molecular Medicine, National Taiwan University, Taipei 100, Taiwan; 10 Graduate Institute of Clinical Medicine, National Taiwan University, Taipei 100, Taiwan

**Keywords:** diabetic cardiomyopathy, emerging therapy, mechanism

## Abstract

Diabetic cardiomyopathy is characterized by abnormal myocardial structure or performance in the absence of coronary artery disease or significant valvular heart disease in patients with diabetes mellitus. The spectrum of diabetic cardiomyopathy ranges from subtle myocardial changes to myocardial fibrosis and diastolic function and finally to symptomatic heart failure. Except for sodium–glucose transport protein 2 inhibitors and possibly bariatric and metabolic surgery, there is currently no specific treatment for this distinct disease entity in patients with diabetes. The molecular mechanism of diabetic cardiomyopathy includes impaired nutrient-sensing signaling, dysregulated autophagy, impaired mitochondrial energetics, altered fuel utilization, oxidative stress and lipid peroxidation, advanced glycation end-products, inflammation, impaired calcium homeostasis, abnormal endothelial function and nitric oxide production, aberrant epidermal growth factor receptor signaling, the activation of the renin–angiotensin–aldosterone system and sympathetic hyperactivity, and extracellular matrix accumulation and fibrosis. Here, we summarize several important emerging treatments for diabetic cardiomyopathy targeting specific molecular mechanisms, with evidence from preclinical studies and clinical trials.

## 1. Introduction: Diabetic Cardiomyopathy as a Distinct Disease Entity

Diabetes mellitus (DM) is an independent risk factor for heart failure (HF), and HF is one of the leading causes of morbidity and mortality in diabetic patients. On the other hand, around 40% of patients with HF with preserved ejection fraction (HFpEF) have diabetes, and diabetes is thought to be closely related to the pathophysiology of HFpEF [1]. Their relationship has been profoundly studied in recent decades.

Diabetic cardiomyopathy is characterized by the existence of an abnormal myocardial structure or performance in the absence of other cardiac lesions, such as coronary artery disease or significant valvular disease, in individuals with DM.

It was first described in 1972 in postmortem pathological findings from four diabetic patients who manifested HF symptoms without evidence of coronary artery or valve disease [2]. The Framingham study showed that HF was two times more likely to develop in men with diabetes and five times more likely to occur in women with diabetes than in nondiabetics after adjustments for age, coronary heart disease, and hypertension in 1974 [3]. This led to the identification of diabetes as an independent risk factor for HF and the recognition of this distinct clinical entity. However, owing to the lack of accepted diagnostic criteria and information on subclinical cardiovascular disease (CVD) in the early stages of DM, the existence of diabetic cardiomyopathy has been debated for many years [4]. New evidence and trials have brought us better insight into diabetic cardiomyopathy, and paradigm-shifting strategies have emerged.

### 1.1. Definition of Diabetic Cardiomyopathy

In 2013, the American College of Cardiology Foundation, the American Heart Association [5], and the European Society of Cardiology, in collaboration with the European Association for the Study of Diabetes [6], defined diabetic cardiomyopathy as a clinical condition of ventricular dysfunction that occurs in the absence of significant coronary artery disease, hypertension, and valvular heart disease in patients with DM.

### 1.2. Evolution of Diabetic Cardiomyopathy to Clinical Heart Failure

The clinical manifestations of diabetic cardiomyopathy range widely from only cellular anomalies to structural remodeling without symptoms and finally to symptomatic heart failure.

Hyperglycemia, hyperinsulinemia, elevated fatty acids, and impaired insulin signaling are the primary initiators of diabetic cardiomyopathy [7]. Subtle myocardial changes develop before symptomatic heart failure. The first stage of diabetic cardiomyopathy is clinically asymptomatic and is characterized by impaired metabolic signaling and altered cardiac energy utilization, leading to myocardial stiffness [8]. Decreased left ventricular (LV) compliance, impaired early diastolic filling, increased atrial filling and enlargement, prolonged isovolumetric relaxation, and elevated left ventricular end-diastolic pressure develop [7]. Cardiac diastolic dysfunction in rodents was associated with impaired cardiac insulin metabolic signaling in a study that observed cine magnetic resonance imaging [9].

In the second stage, structural remodeling, such as left ventricular hypertrophy and fibrosis, is detected by clinical imaging studies, without typical HF symptoms [10]. A community-based study of 6814 people without coronary artery disease demonstrated that increasing indices of metabolic syndrome led to two thirds of patients developing HF with a normal ejection fraction after 4 years of follow-up [11].

If left untreated, apparent HF will develop. The third stage of diabetic cardiomyopathy is characterized by further anatomical or functional decline [7]. With the progression of diabetic cardiomyopathy, diastolic dysfunction may coexist with systolic dysfunction [7]. In the Cardiovascular Health Study involving 5201 people, the ventricular septal and left posterior myocardial walls were found to be thicker in diabetic patients than in nondiabetic individuals, which was associated with compromised systolic or diastolic functions [12].

### 1.3. Epidemiology of Diabetic Cardiomyopathy

The prevalence of type 2 diabetes (T2D) has reached a pandemic scale. According to an International Diabetes Federation (IDF) report, there are 451 million people with diabetes worldwide. Furthermore, almost half of all people living with diabetes are undiagnosed [13]. The Framingham Heart Study found that the incidence of HF was increased in both male and female diabetic patients compared with age-matched nondiabetic individuals. This association was independent of obesity, hypertension, dyslipidemia, and coronary heart disease [3] and was observed across different genders, different ethnic subgroups, and the presence or absence of hypertension or obesity [14].

Further data derived from a population-based observational study in the Cardiovascular Health Study, the Strong Heart Study [15], and the Multi-Ethnic Study of Atherosclerosis (MESA) [16] demonstrated significant differences in LV mass and wall thickness as well as increased diastolic and systolic dysfunctions between diabetic patients and normal individuals. The incidence of HF was higher in diabetic (39%) compared to nondiabetic (23%) patients, with a relative risk of 1.3 for developing HF [17].

In type 1 DM, each 1% increase in glycated hemoglobin A1C was linked to a 30% increase in the risk of HF [18]. In type 2 DM, each 1% rise in hemoglobin A1C levels was associated with an 8% increase in risk, independent of other risk factors [19]. In recently conducted large randomized control trials (RCT) of diabetic treatments, 8–10% of diabetic patients were hospitalized for HF [20,21]. Furthermore, 50% of registered HF with a reduced left ventricular ejection fraction (LVEF) (HFrEF) patients have diabetes [22,23]. In another RCT focused on HF patients with preserved LVEF (HFrEF), 49% were diabetic [24].

Among subjects with diabetic cardiomyopathy, the cumulative probability of death was 18% and the probability of developing HF was 31% at 9 years. Diabetic cardiomyopathy is relatively common in the community, with a prevalence of 1.1% [25].

### 1.4. Initial and Serial Evaluation

The initial evaluation starts from the status of diabetes. After confirming the diagnosis of diabetes with the hemoglobin A1c and glucose levels, the American Diabetes Association recommends screening the cardiac function of patients with type 2 DM to prevent diabetic cardiomyopathy. An echocardiogram is a safe and efficient tool to access cardiac function and to exclude other causes of heart failure, such as valvular heart disease. Further studies to exclude other causes of HF, such as cardiovascular disease and other types of cardiomyopathy, should be part of the treatment plan. Computed tomography and cinematic magnetic resonance imaging have been applied to detect changes in cardiac structure (i.e., fibrosis) and function [7]. Although many biomarkers have been evaluated in experimental studies, they were not cost-effectively available for clinical scenarios.

## 2. Current Treatment Strategies for Heart Failure in Patients with Diabetes

First of all, optimal glycemic control has demonstrated a benefit. In the UKPDS (UK Prospective Diabetes Study), a 1% reduction in hemoglobin A1C was associated with a 16% risk reduction for the development of HF [20]. The glycemic target depends on the existence of comorbidity [26].

Implementing healthy behaviors such as nutrition therapy, aerobic exercise, weight reduction, and smoking cessation is an effective therapeutic approach for preventing diabetic cardiomyopathy [27]. In the Standards of Care in Diabetes of The American Diabetes Association of 2023, the evidence level was A [28].

Further drug treatments, devices, and surgeries provide more options for people with diabetes. Their effects on HF are summarized as follows:

### 2.1. Sodium–Glucose Cotransporter 2 Inhibitors (SGLT2i)

Cardiorenal outcome trials of SGLT2i have clearly demonstrated their efficacy in reducing the risk of major adverse cardiac events (MACE, including myocardial infarction, stroke, and cardiovascular death), hospitalization for heart failure, and all-cause mortality in individuals with type 2 diabetes [29,30]. Several RCTs in patients with type 2 diabetes and either established CVD or a high risk for CVD have shown that SGLT2i prevent HF hospitalizations [20,21]. In the Standards of Care in Diabetes guideline of the American Diabetes Association of 2023, SGLT2i were recommended as the first choice for diabetic patients with HD (evidence level: A) [28].

A 31% reduction in overall HF hospitalizations was noted, irrespective of the presence or absence of pre-existing HF, although only 10% to 14% of participants had HF at baseline. The benefit was independent of the glucose-lowering effects [31]. The mechanism of the cardioprotective effect of SGLT2i, independent of the glucose-lowering effect, remains to be determined and is currently an active area of research [32,33].

### 2.2. Glucagon-like Peptide-1 Receptor Agonists (GLP-1 RAs)

Beyond glycemic control, GLP-1 RAs have also been approved for reducing the risk of MACE in patients with type 2 diabetes with established CVD (dulaglutide, liraglutide, and subcutaneous semaglutide) or multiple cardiovascular risk factors (dulaglutide) and for weight reduction (subcutaneous liraglutide and semaglutide) [34,35].

No clear benefit for heart failure in diabetic patients was observed in RCTs, including the LEADER trial (Liraglutide Effect and Action in Diabetes: Evaluation of Cardiovascular Outcome Results) [36], the CARMELINA trial (Cardiovascular and Renal Microvascular Outcome Study With Linagliptin) [37], SUSTAIN-6 (Trial to Evaluate Cardiovascular and Other Long-Term Outcomes with Semaglutide in Subjects with Type 2 Diabetes) [38], the ELIXA trial (Evaluation of Lixisenatide in Acute Coronary Syndrome) [39], and the REWIND trial (Researching Cardiovascular Events with a Weekly Incretin in Diabetes) [40].

In contrast, the HARMONY Outcomes trial (Albiglutide and Cardiovascular Outcomes in Patients With Type 2 Diabetes and Cardiovascular Disease), which specifies HF hospitalization as a secondary endpoint, showed a significant 29% reduced risk of HF hospitalization with GLP-1 RAs [41]. In a meta-analysis of the seven RCTs, GLP-1 RAs showed statistically significant 9% reductions in the risk of HF hospitalization [42]. Although GLP-1 RAs may theoretically benefit patients with HFpEF, no RCT has specifically investigated GLP-1 RAs in diabetic patients with HFpEF. The effect of GLP-1 RAs on HF might need to be investigated separately in patients with HFrEF and HFpEF. Therefore, the Standards of Care in Diabetes of the American Diabetes Association in 2023 only recommended GLP-1RAs as one of the first choices for diabetic patients with atherosclerotic cardiovascular disease (evidence level: A) but not for those with HF [43].

### 2.3. Metformin

In the United Kingdom Prospective Diabetes Study (UKPDS 34), overweight type 2 diabetic patients receiving an intensive metformin treatment showed a 39% reduction in the risk of myocardial infarction and a 36% reduction in death from any cause [43]. In a retrospective observational study of 10,920 patients, metformin use was associated with low mortality risk in diabetic individuals with HF [44]. However, a meta-analysis of 13 RCTs involving 13,110 patients did not support a beneficial effect of metformin on HF [45]. There is no specific recommendation for metformin in diabetic patients with HF in the current guidelines.

### 2.4. Dipeptidyl Peptidase 4 Inhibitors (DPP-4i)

Cardiovascular outcome trials have demonstrated the cardiovascular safety without risk reduction of four DPP-4i (saxagliptin, alogliptin, sitagliptin, and linagliptin) [46]. While generally well tolerated, an increased risk of HF hospitalization was found with saxagliptin [47]. Whether the risk of worsening HF is a class effect of DPP-4i is unclear. The Consensus Report of the American Diabetes Association in 2022 recommended that DPP-4i should be avoided in diabetic patients with stage B and C HF [48].

### 2.5. Sulfonylurea

In the UKPDS 33 trial comparing intensive glycemic control using sulfonylurea or insulin to conventional therapy, the result showed no difference in the incidence of HF in newly diagnosed T2D patients (HR, 0.91; 95% CI, 0.54–1.52) [49].

However, sulfonylurea was associated with an increased risk of death in patients with T2D and HF in observational studies [50,51,52]. The Consensus Report of the American Diabetes Association in 2022 recommended that sulfonylurea should be avoided in diabetic patients with stage B and C HF [48].

### 2.6. Insulin

The Diabetes Control and Complications Trial/Epidemiology of Diabetes Interventions and Complications (DCCT/EDIC) study for type 1 diabetes reported a risk reduction in microvascular and macrovascular complications with an intensive insulin treatment [53]. However, an RCT of 24,012 patients showed that insulin treatment worsened HF in diabetic patients with chronic HF [54]. There is no specific recommendation for insulin therapy in diabetic patients with HF in the current guidelines.

### 2.7. Thiazolidinediones (TZDs)

TZDs increase insulin sensitivity by activating nuclear peroxisome proliferator-activated receptor gamma (PPAR-γ). PPAR-γ increases sodium reabsorption in the collecting ducts of the kidney and causes fluid retention. TZDs have been associated with increased rates of HF in RCTs of patients with type 2 diabetes who were free of symptomatic HF at baseline. Therefore, TZDs should be avoided in patients with reduced LVEF [55,56]. The Consensus Report of the American Diabetes Association in 2022 recommended that TZDs should be avoided in diabetic patients with stage B and C HF [48].

## 3. Molecular Mechanism of Diabetic Cardiomyopathy

In addition to the influence of comorbidities such as hypertension and obesity, the diabetic condition *per se*, including hyperglycemia, insulin resistance, and hyperinsulinemia contribute to diabetic cardiomyopathy because diastolic dysfunction is often observed in type 1 diabetes [7]. Cardiomyocyte-based changes are essential at the beginning of the disease, whereas extracellular matrix (ECM)-based changes become prominent in the advanced stages [57].

### 3.1. Impaired Nutrient-Sensing Signaling, Dysregulated Autophagy, Impaired Mitochondrial Energetics, Metabolic Alteration, and Altered Fuel Utilization

**Figure 1:**
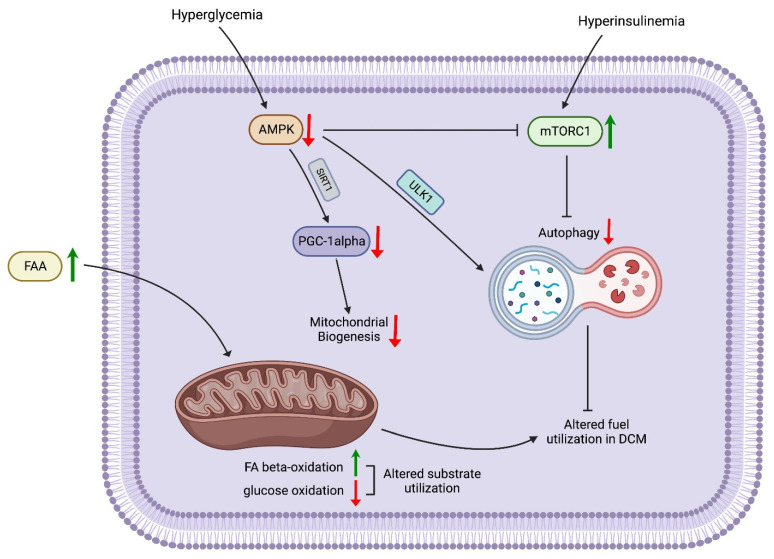
Impaired nutrient-sensing signaling, dysregulated autophagy, impaired mitochondrial energetics, and altered fuel utilization in the pathogenesis of diabetic cardiomyopathy. AMPK:5’ adenosine monophosphate-activated protein kinase; mTORC1: mammalian target of rapamycin complex 1; ULK1: unc-51-like autophagy activating kinase 1; PGC-1á: peroxisome proliferator-activated receptor-gamma coactivator 1-alpha; FFA: fatty acid, DCM: diabetic cardiomyopathy.

Hyperinsulinemia develops early, even in prediabetes. Insulin is a potent activator of the nutrient-sensing mammalian target of the rapamycin (mTOR) pathway, which regulates protein translation, cell growth and proliferation, and autophagy [7]. Preclinical studies showed that activating the mTORC1 pathway promotes diabetic cardiomyopathy or cardiac hypertrophy [58,59], while inhibiting the mTORC1 pathway prevents them [60] (Figure 1).

Hyperglycemia develops in overt diabetes. The AMP-activated protein kinase (AMPK) pathway senses an energy deficiency and modulates cellular metabolism, primarily to activate the uptake and oxidation of glucose and fatty acid [61]. Hyperglycemia has been shown to inhibit AMPKα2, leading to the development of diabetic cardiomyopathy [62].

The AMPK pathway also promotes autophagy [63]. Autophagy is a catabolic process induced by cellular stress, such as a nutrient shortage, which recycles damaged organelles to prevent further cell damage. The recycled organelles can serve as an energy source and as building blocks for cells [61]. The downregulated AMPK pathway in diabetes decreased cardiac autophagy, causing cardiac dysfunction in a diabetic cardiomyopathy mouse model [63]. Cardiac-specific AMPK dominant-negative repression decreased cardiac autophagy, exacerbated cardiac dysfunction, and increased mortality in diabetic mice, which was reversed by metformin, an activator of AMPK [64]. In addition, the AMPK pathway inhibits the mTORC1 pathway [7]. The activation of the AMPK pathway was shown to inhibit cardiac hypertrophy by promoting autophagy through inhibiting mTORC1 [65] (Figure 1).

Furthermore, the AMPK pathway stimulates mitochondrial biogenesis through peroxisome proliferator-activated receptor-gamma coactivator-1 alpha (PGC-1α)-dependent transcriptional control and promotes mitophagy [61]. Mitochondrial dysfunction plays a central role in developing diabetic cardiomyopathy [66]. High glucose and fatty acid fluxes overload the mitochondrial electron transfer chains, leading to increased mitochondrial proton leak, increased emission of reactive oxygen species (ROS), mitochondrial oxidative damage, attenuated oxidative phosphorylation, and decreased ATP production, which is required for myocardial relaxation [67].

Impaired insulin receptor signaling and the insulin-like growth factor 1 (IGF-1) receptor also contribute to cardiac fibrosis. Transgenic cardiac-specific IGF-1 receptor expression protects against cardiac fibrosis and diastolic dysfunction in diabetic cardiomyopathy [68]. An IGF-1 treatment reversed diabetic cardiomyopathy in diabetic rats [69].

In diabetes, triglycerides and circulating free fatty acids are elevated [70]. Under normal conditions, both free fatty acids and glucose are substrates for cardiomyocytes to generate ATP, with free fatty acids being the predominant one. Insulin resistance develops early in diabetes, with reduced glucose uptake of cardiomyocytes. Therefore, the energy substrate utilized by myocardial mitochondria shifts to fatty acids [71]. Furthermore, the increased utilization of triglycerides and their intermediate metabolites, such as ceramides, in cardiomyocytes causes myocardial lipotoxicity [72] (Figure 1).

Myocardial ketone body utilization is suppressed in diabetes, possibly due to an impaired mitochondrial function where ketone bodies are converted to acetyl-CoA [73]. The loss of this ketone utilization capability impairs myocardial function. A cardiomyocyte-specific knockout of succinyl-CoA:3 ketoacid-CoA transferase, the key enzyme for the terminal step of ketone body oxidation, leads to cardiomegaly [74]. Interestingly, SGLT2 inhibitors increase ketone levels, thus providing a more efficient energy source for patients with diabetic HF [75].

### 3.2. Oxidative Stress, Lipid Peroxidation, Advanced Glycation End-Products (AGEs), and Maladaptive Immune Responses

In overt diabetes, cells are exposed to high glucose and fatty acid levels, which overloads the electron transfer chain of mitochondria with increased emission of their byproduct superoxide, mainly from complexes I and III. Superoxide is converted to ROS, causing oxidative damage. The central importance of ROS is demonstrated by the fact that this can be prevented when ROS generation is eliminated [66]. In addition, excessive glucose flux also leads to the overproduction of glycolysis intermediates, which triggers multiple pathogenic signaling pathways, including the methylglyoxal (MG)/advanced glycation end-products (AGEs) pathway, the hexosamine pathway, protein kinase C signaling, and the aldose reductase/sorbitol polyol pathway [66]. Cardiac-specific overexpression of the protein kinase C β2 isoform resulted in cardiac hypertrophy and fibrosis. In contrast, treatment with LY333531, a protein kinase C β isoform inhibitor, prevented these changes [76], demonstrating the importance of these upregulated pathways in diabetes. Mice with a double knockout of PKCδ and PKCε developed cardiomyocyte hyperplasia and ventricular stiffening [77].

Lipid peroxidation is triggered by ROS attacking the polyunsaturated fatty acids of the cell membrane, leading to the generation of reactive toxic aldehydes such as 4-hydroxy-2-nonenal (4-HNE), malondialdehyde, and acrolein. These aldehydes launch electrophilic attacks and adduct to proteins, causing cardiac damage [78,79,80]. Aldehyde dehydrogenase 2 (ALDH2), located in mitochondria, is the major enzyme for detoxifying these toxic aldehydes [78,79,80]. We demonstrated reductions in the expression level and enzymatic activity of ALDH2 in diabetic hearts. The ALDH2 activator AD-9308 improved cardiac diastolic and wall remodeling in diabetic mice [81].

**Figure 2:**
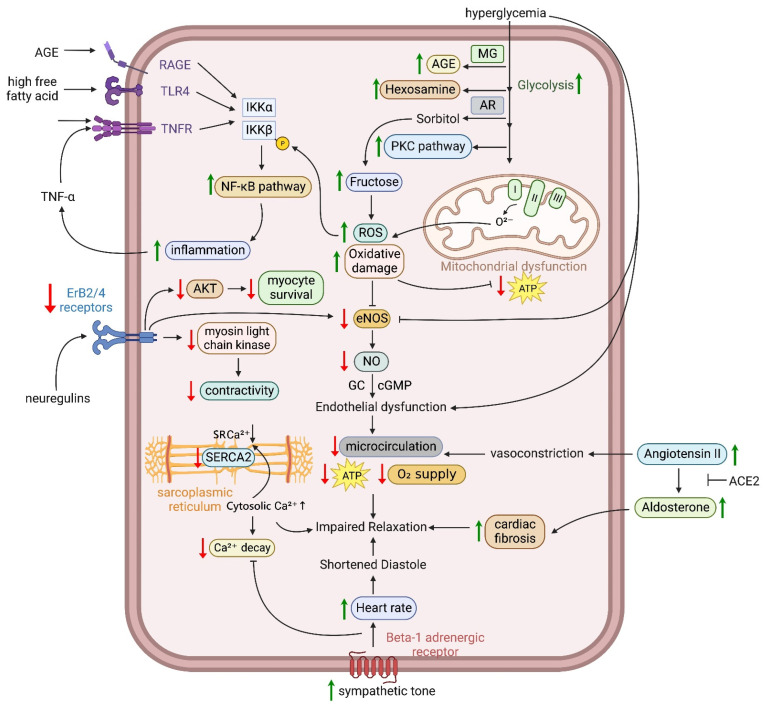
Oxidative stress, inflammation, impaired calcium homeostasis, abnormal endothelial function and nitric oxide production, aberrant epidermal growth factor receptor singling, and activation of the renin–angiotensin–aldosterone and sympathetic systemsin the pathogenesis of diabetic cardiomyopathy. MG: methylglyoxal, AGE: advanced glycation end-products, AR: aldose reductase, PKC: protein kinase C; ROS: reactive oxygen species; RAGE: receptor for advanced glycation end-products; TRL4: toll-like receptor 4; TNFR: tumor necrosis factor receptor; NF-κB: nuclear factor kappa-B; TNF-α: tumor necrosis factor-α; GC: guanylate cyclase; eNOS: constitutive nitric oxide synthase; ErbB2/4 (v-erb-b2 avian erythroblastic leukemia viral oncogene homolog 2); NOX: NADPH oxidase; ACE2: angiotensin-converting enzyme 2; SERCA2a: sarcoplasmic reticulum Ca^2+^-ATPase 2a; NO: nitric oxide; ATP: adenosine triphosphate, IKK: IκB kinase; SR: sarcoplasmic reticulum.

Elevated fatty acids and hyperglycemia activate the NACHT, LRR, and PYD domains-containing protein 3 (NLRP3) inflammasome in endothelial cells [82]. Furthermore, ROS, AGEs, and fatty acids directly induce nuclear factor kappa-B (NF-κB) activation through receptor binding, triggering inflammatory responses. Inflammatory cytokine expression was also found to be elevated in diabetic hearts [83,84]. Tumor necrosis factor-α (TNF-α) provoked cardiomyocyte hypertrophy [83], while overexpressing C-reactive protein exacerbated left ventricular remodeling in diabetic mice [84]. However, inhibition of the p38 mitogen-activated protein kinase (MAPK) stress pathway reduced the inflammatory cytokines TNF-α, IL6, and IL1-β in diabetic hearts and attenuated left ventricular dysfunction in diabetic cardiomyopathy [85] (Figure 2).

### 3.3. Impaired Calcium Homeostasis

At rest, the cytosolic calcium (Ca^2^) concentration of cardiomyocytes is maintained at a low level by the sarcoplasmic reticulum Ca^2+^-ATPase 2a (SERCA2a), which takes up cytosolic Ca^2+^ into the sarcoplasmic reticulum (SR). Electric stimulation depolarizes the cardiomyocyte membrane and opens the slow L-type Ca^2+^ channels, leading to a small flux of Ca^2+^, which in turn triggers a large Ca^2+^ flux from the SR to the cytosol via the ryanodine receptor 2 (RYR2) to trigger cardiac contraction. Cardiac relaxation occurs when cytosolic Ca^2+^ is taken up again by SERCA2a into the SR or, to a lesser extent, transported outside the cells by the Na^+^/Ca^2+^ exchanger (NCX).

Previous studies in diabetic rodents showed either no changes [86,87,88,89] or a reduction in the L-type Ca^2+^ current [90,91,92,93,94,95] in cardiomyocytes. In *db/db* mice and Zuker fat diabetic rats, the L-type Ca^2+^ current was reduced in cardiomyocytes [96,97], which was associated with impaired systolic function.

RyR2 dysfunction contributes to cardiac hypertrophy, polymorphic ventricular tachycardia, or arrhythmogenic right ventricular cardiomyopathy [98,99,100]. In several diabetic rodent models, the expression of RyR2 was reduced [89,101,102,103]. In diabetic rats, the release of Ca^2+^ from the SR was reduced and the time to peak Ca^2+^ transients was delayed [88,104,105,106]. Furthermore, feeding a high-fat diet to mice led to increased arrhythmic episodes, which were associated with an enhanced RyR2 response to Ca^2+^ through redox modifications [107].

The reduced SERCA2a activity observed in diabetic hearts led to the prolongation of cytosolic Ca^2+^ decay, prolonged diastolic relaxation, and impaired diastolic function [108]. Pharmacological stimulation of SERCA2a improved intracellular Ca^2+^ handling and diastolic dysfunction in a model of diabetic cardiomyopathy [109].

Na^+^-H^+^ exchanger isoform 1 (NHE1) is responsible for the balance of intracellular acidity. The activity of NHE1 is low in the basal condition but is highly activated during myocardial ischemia to prevent intracellular acidosis, resulting in increased Na^+^ entry into the cell [110]. The increased intracellular Na^+^ concentration causes cytosolic Ca^2+^ accumulation in cardiomyocytes via the NCX Na^+^/Ca^2+^ exchanger. Chronic accumulation of cytosolic Ca^2+^ has been implicated in the development of cardiac hypertrophy and arrhythmias [111]. Transgenic mice overexpressing NHE1 in the heart developed cardiac hypertrophy, contractile dysfunction, and heart failure [112,113].

Phospholamban binds and inhibits SERCA2a, and the phosphorylation of phospholamban relieves this inhibition, leading to enhanced SRER2a activity and an increased SR Ca^2+^ concentration. The SR Ca^2+^ load was also enhanced via Ca^2+^/calmodulin-dependent protein kinase II (CaMKII)-dependent phosphorylation of phospholamban in HNE1 transgenic mice. In addition, the activity of calcineurin and CaMKII was also highly increased in NHE1 transgenic mice [112]. Previous studies showed that the overexpression of calcineurin [114] in the heart caused cardiac hypertrophy and heart failure, while the overexpression of CaMKII caused cardiac hypertrophy, arrhythmias, and sudden death [115].

In *ob/ob* diabetic mice with cardiac hypertrophy, the NHE1 activity in isolated cardiomyocytes was enhanced. The enhanced myocardial NHE1 activity increased cytosolic Na ^+^ and subsequently cytosolic Ca^2+^, leading to an altered mitochondrial Ca^2+^ content and an altered membrane potential, which were minimized by NHE1 inhibition [116]. In Goto–Kakizaki diabetic rats with hypertrophic left ventricles, enhanced NHE1 activity and increased cytosolic Ca2^+^ were observed, which were associated with ventricular myocyte hypertrophy. Several molecular pathways related to NHE1, including the phosphorylation of extracellular signal-regulated protein kinase (Erk), Akt, and the Ca^2+^/calmodulin-dependent kinase, were also elevated in isolated myocytes of Goto–Kakizaki diabetic rats. A chronic treatment with the NHE1 inhibitor cariporide normalized the NHE1 activity, decreased the cytosolic Ca2^+^ levels, and reduced the LV myocyte hypertrophy [117]. Interestingly, SGLT2 inhibitors inhibit NHE1 activity, reduce cardiac cytosolic Na^+^ and Ca^2+^, and cause coronary relaxation [118] (Figure 2).

### 3.4. Abnormal Endothelial Function and Nitric Oxide (NO) Production

Hyperglycemia is widely accepted to cause endothelial dysfunction via altered oxidative damage. The high muscle-to-capillary ratio in the heart (approximately 1:1) indicates the importance of cardiac microcirculation [119]. Physiologically, vasoactive substances, including NO and prostacyclin, exert beneficial vasodilatory effects on myocardial microcirculation.

NO is synthesized from L-arginine by endothelial nitric oxide synthase (eNOS) in the endothelium and the endocardium. It activates the soluble enzyme guanylate cyclase in vascular smooth muscle cells or cardiomyocytes, which catalyzes the conversion of guanosine triphosphate into cyclic guanosine monophosphate (cGMP). cGMP decreases the intracellular free calcium levels and activates cGMP-dependent protein kinase, resulting in the relaxation of smooth muscle cells or cardiomyocytes [120,121].

Insulin enhances constitutive eNOS expression in endothelial cells [122,123], while hyperglycemia and AGEs reduce eNOS activity [124,125]. Reduced NO production increases intracellular Ca^2+^ and reduces myocardial [126] and endothelial sarcoplasmic reticulum Ca^2+^ uptake, leading to impaired cardiac relaxation and vascular stiffness [127]. Significant dysfunction of the microcirculation develops in diabetic hearts due to reduced NO [128]. In addition, a recent investigation showed that endothelial-cell-derived endothelin-1, a vasoconstrictor, promotes cardiac fibrosis and diastolic dysfunction in diabetic patients [129]. Furthermore, abnormal cardiac microcirculation and relaxation contributes to the progression of diabetic cardiomyopathy [125] (Figure 2).

### 3.5. Aberrant Epidermal Growth Factor Receptor (EGFR)-v-erb-b2 Avian Erythroblastic Leukemia Viral Oncogene Homolog 2(ErbB2/4) Receptor Signaling

ErbBs, including ErbB1-4, are tyrosine kinase receptors. After ligands bind to the ErbB family of receptors, they trigger the ErbB signaling pathways, which involve cell growth, differentiation, migration, adhesion, and survival [130]. ErbB2/ErbB4 receptor expression is reduced in rats with diabetic cardiomyopathy [131]. Abundant evidence has shown that the activation of ErbB2 (also named HER2/neu) or ErbB4 prevents cardiomyopathy [132,133], the while inhibition of ErbB2 or ErbB4 causes heart failure [134,135,136,137], possibly through the modulation of Akt, eNOS, and myosin light chain kinase, an enzyme critical for cardiac muscle contraction [138] (Figure 2).

### 3.6. Activation of Renin–Angiotensin–Aldosterone System (RAAS) and Autonomic Dysfunction

Hyperglycemia increases angiotensinogen and angiotensin II production by activating p53 [139,140,141]. Serum angiotensin II levels correlate significantly with postprandial glucose concentrations [142]. In addition to the well-established effect of RAAS activation on pressure and volume overload in the myocardium, angiotensin II directly increases vascular ROS production via the activation of NADPH oxidase (NOX)and reduces endothelial NO production [143]. The proinflammatory angiotensin II receptor 1 is also upregulated in diabetes, which increases endothelial leukocyte adhesion, proinflammatory cytokine expression, and macrophage infiltration with M1 polarization in the myocardium [144]. Furthermore, aldosterone also directly causes myocardial fibrosis [9,145].

Angiotensin-converting enzyme 2 (ACE2) and its product, angiotensin 1–7, are negative regulators of the RAAS. Consistently, angiotensin 1–7 ameliorated diabetic cardiomyopathy and diastolic dysfunction in *db/db* mice by reducing myocardial lipid accumulation and improving glucose oxidation [146].

Autonomic neuropathy is associated with diastolic dysfunction and HFpEF in patients with type 2 or type 1 diabetes [147,148]. Autonomic neuropathy usually manifests a predominance of (particularly nocturnal) sympathetic activity. Activating the sympathetic nervous system enhances β-1 adrenergic receptor signaling, promotes cardiac hypertrophy and interstitial fibrosis, diminishes the coronary blood flow reserve, and leads to diastolic dysfunction [149].

In addition, echocardiographic studies have revealed a significant correlation between the severities of autonomic neuropathy and reduced diastolic filling in normotensive type 1 diabetic patients [150]. Diabetic patients with autonomic dysfunction have a higher resting heart rate than nondiabetic patients, which reduces coronary blood flow with a shortened diastole [151] (Figure 2).

### 3.7. Extracellular Matrix (ECM) Accumulation and Fibrosis

The final and probably irreversible stage of diabetic cardiomyopathy is cardiac fibrosis. Insulin signaling modulates cardiac fibrosis. A cardiac-specific knockout of insulin receptors attenuated cardiac fibrosis and hypertrophy in response to pressure overload via transverse aortic constriction [152]. Consistently, a cardiac-specific knockout of insulin receptor substrate 1 (IRS1) has prevented cardiac hypertrophy and fibrosis [153]. The muscle-specific E3 ligase mitsugumin 53 (MG53) negatively regulates insulin signaling through the proteasomal degradation of insulin receptors and IRS1 [154]. Hyperglycemia or hyperinsulinemia induces MG53 secretion, and MG53 is upregulated in humans and rodents with diabetes [155]. MG53 transgenic mice developed severe diabetic cardiomyopathy [156], while a MG53 E3 ligase-dead mutation protected against diabetic cardiomyopathy [157], suggesting that chronic hyperinsulinemia promotes cardiac fibrosis.

A hyperglycemia-flux-induced generation of glycolysis intermediates provokes AGE accumulation and the hexosamine pathway. AGEs promote collagen crosslinking and cause myocardial stiffness [158]. O-GlcNAc transferase (OGT) is an enzyme for the reversible post-translational modification of serine and threonine residues of proteins, including phospholamban, calmodulin kinase II, and troponin I. The hyperglycemia-induced hexosamine pathway supplies the N-acetylglucosamine moiety (O-GlcNAc), which is O-linked to these proteins by OGT. Thus, chronic activation of the hexosamine pathway in diabetic hearts affects Ca^2+^ handling, the contractile properties, and ventricular hypertrophy [159]. The SGLT2 inhibitor dapagliflozin has recently been shown to lower cardiac hexosamine flux and reduces *O*-GlcNAcylated protein levels, thus preventing diabetic cardiomyopathy [160].

ROS-activated stress pathways, such as the c-Jun N-terminal kinase (JNK) pathway, AGE/RAGE accumulation, and the activated PKC pathway, and activated inflammatory pathways, such as the p83 mitogen-activated protein kinase (MAPK) and extracellular signal-regulated kinase (ERK) pathways all increase collagen production and ECM accumulation through the transforming growth factor β1 (TGFβ1) pathway [161,162]. Toxic aldehydes derived from lipid peroxidation crosslink cytoskeletons and connective tissue, while the activation of ALDH2 protects against cardiac fibrosis [163].

In addition, reduced NO bioavailability causes titin phosphorylation and stiffening from titin N2B isoform expression, leading to cardiac stiffness through the activation of the NO–cyclic guanosine monophosphate (cGMP)–protein kinase G (PKG) pathway. Titin is a giant sarcoplasmic filament that is responsible for sarcomere stiffness. The stiffness maintains the structural integrity of the contracting sarcomere [164]. Reduced NO bioavailability also increases collagen crosslinking, leading to cardiac fibrosis.

**Figure 3:**
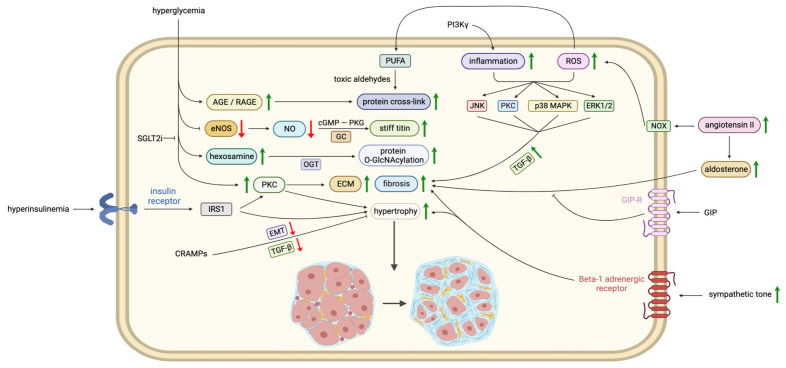
The pathogenesis of extracellular matrix (ECM) accumulation and fibrosis in diabetic cardiomyopathy.PI3Kã:phosphoinositide 3-kinaseã; ROS: reactive oxygen species; JNK: c-Jun N-terminal kinase, PKC: protein kinase C; p38 MAPK: p38 mitogen-activated protein kinase; GC: guanylate cyclase; cGMP: cyclic guanosine monophosphate; PKG: protein kinase G; ERK1/2: extracellular signal-regulated protein kinase 1/2;TGF-β: transforming growth factor-beta; GIP: gastric inhibitory polypeptide; GIP R: gastric inhibitory polypeptide receptor; IRS-1: insulin receptor substrate 1; O-GlcNAcylation (O-linked-N-acetylglucosaminylation); EMT: epithelial–mesenchymal transition; OCT: O-linked N-acetylglucosamine transferase; SGLT2i: sodium–glucose cotransporter 2inhibitors: NOX: NADPH oxidase; PUFA: polyunsaturated fatty acid; ECM: extracellular matrix.

Furthermore, NO regulates the function of tissue transglutaminase 2 in the vasculature [165]. Tissue transglutaminase 2 is abundantly expressed in the myocardium and regulates vascular permeability, contractile activity, collagen crosslinking, and injury response [166]. It is upregulated in diabetic cardiomyopathy [167]. Tissue transglutaminase 2 knockout mice displayed less cytoskeletal remodeling, collagen assembly, and stiff vasculature, with attenuated matrix remodeling in the heart [166]. Cardiac-specific overexpression of transglutaminase 2 results in myocardial hypertrophy [168] (Figure 3).

### 3.8. Epigenetic Changes in Diabetic Cardiomyopathy

Several cases of hypermethylation or demethylation, histone modifications, and altered expression of noncoding RNA associated with diabetic cardiomyopathy in diabetic patients or rodents have been reported and reviewed elsewhere [169,170,171]. Here, we summarize the recent progress with therapeutic potential.

The protein p66^Shc^ is a key regulator of inflammation and ROS production, which was upregulated in the hearts of diabetic mice. Demethylation and histone H3 acetylation of the promoter of p66^Shc^ caused its persistent transcription, leading to increased cardiac oxidative stress and inflammation and ventricular dysfunction. Importantly, the silencing of p66Shc in diabetic mice restored cardiac function [172].

In another study, the expression of JunD (JunD proto-oncogene subunit), a member of the AP-1 (activator protein-1) family, was decreased in the hearts of both streptozotocin-induced diabetic mice and diabetic patients. Consistently, the methylation of the JunD promoter was enhanced in the hearts of streptozotocin-induced mice. The methylation was accompanied by repressive H3K9me3 marks of histone H3 at the JunD promoter. In addition, the expression of MEN1, a repressor of JunD, was increased in the hearts of diabetic mice. A high-glucose treatment increased MEN1 and decreased JunD expression in cardiomyocytes. Furthermore, miRNA profiling of diabetic hearts identified that miRNA-673, which suppresses MEN1, was downregulated. Overexpressing miRNA-673 restored the changes in the expression of MEN1 and JunD induced by hyperglycemia. Furthermore, cardiac-specific overexpression of JunD protects against diabetic cardiac dysfunction by modulating the expression of the genes involved in ROS scavenging and generation. Interestingly, histone deacetylase (HDAC) inhibitors have been shown to preserve cardiac performance and suppress cardiac remodeling in murine diabetic cardiomyopathy [173].

A study showed that a knockdown of miR-451 using adenovirus shmiR-451 attenuated cardiac fibrosis and improved cardiac function in streptozotocin-induced diabetic mice. Inhibition of miR-451 using the miR-451 antagomir attenuated the hyperglycemia-induced endothelial-to-mesenchymal transition (EMT) in mouse heart endothelial cells, which was abrogated by the inhibition of AMPKα1 [174]. Another miRNA profile of *db/db* mice identified that miR-320 was associated with diabetic cardiomyopathy. The overexpression of miR-320 exacerbated cardiomyopathy, while the inhibition of miR-320 improved the cardiomyopathy of *db/db* mice. Surprisingly, miR-320 activated the transcription of the CD36 fatty acid translocase, leading to increased cardiac fatty acid uptake and lipotoxicity in the heart [175]. These studies exemplify the therapeutic potential of delivering miRNAs to treat diabetic cardiomyopathy.

## 4. Staging and Diagnostic Algorithm of Diabetic Cardiomyopathy

According to our current understanding, we summarize the morphological features, functional changes, and cellular and metabolic abnormalities of each stage of diabetic cardiomyopathy in Table 1 [72,176,177].

There are no appropriate diagnostic strategies for recognizing the early stage of diabetic cardiomyopathy. However, it is important to detect the early stage of diabetic cardiomyopathy in order to initiate some emerging therapies targeting the mechanisms of the early phase to avoid irreversible myocardial damage and prevent disease progression. Cardiac biomarkers would be helpful in the early recognition of diabetic cardiomyopathy. Amongst the conventional and established cardiac biomarkers, N-terminal pro-brain natriuretic peptide is limited to patients with obvious diastolic dysfunction and symptomatic HF [178], and its utility in early-stage diabetic cardiomyopathy is limited. Meanwhile, troponin-I and -T are not specific markers of cardiomyopathy. Numerous novel circulating biomarkers have been identified for diagnosing diabetic cardiomyopathy. However, most of them were from experimental studies or are under investigation. We summarize the promising biomarkers and possible candidates in Table 2.

**Figure 4:**
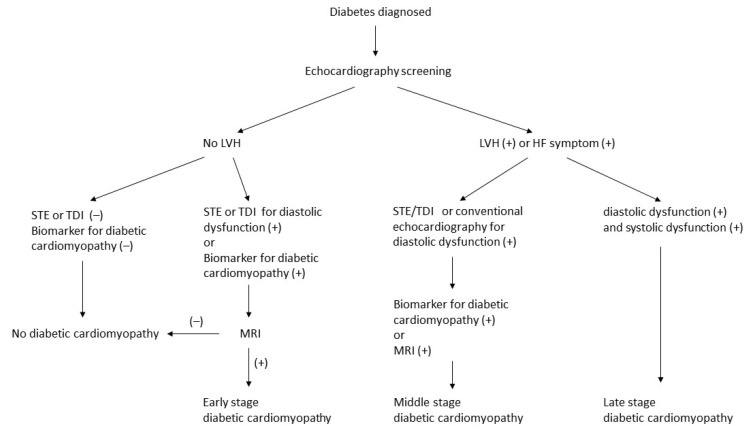
Proposed algorithm for diagnosis of diabetic cardiomyopathy. LVH: left ventricular hypertrophy; MRI: magnetic resonance imaging; STE: speckle-tracking echocardiography; TDI; tissue Doppler imaging.

We propose a diagnostic algorithm for diabetic cardiomyopathy using a combination of echocardiogram, magnetic resonance imaging, and these promising biomarkers to detect and treat this disease entity early on Figure 4.

## 5. Emerging Treatment Targeting Specific Mechanisms of Diabetic Cardiomyopathy

Although the mechanism of diabetic cardiomyopathy has been extensively explored, there are currently no approved or specific therapies. Here, we summarize important emerging therapies that are under investigation.

### 5.1. Modulators of Energy Utilization

Trimetazidine is an anti-ischemic agent [204]. It selectively inhibits long-chain 3-ketoacyl coenzyme A thiolase, an enzyme required for fatty acid oxidation. Its action is attributed to its effect on energy utilization. Trimetazidine shifts energy utilization from free fatty acid oxidation to glucose oxidation in a stressed heart [205,206]. In addition, trimetazidine also triggers autophagy [207], inhibits cardiac fibrosis [208], and prevents cardiomyocyte apoptosis [209,210]. Numerous clinical trials with small sample sizes have demonstrated the efficacy of trimetazidine in the treatment of ischemic or nonischemic heart failure. It improved the left ventricular ejection fraction, symptoms, exercise capacity, and cardiovascular events [211,212]. Owing to its effect on energy utilization, trimetazidine may be more effective in patients with diabetic cardiomyopathy. In a type 2 diabetic rat model, early administration of trimetazidine improved metabolic disturbances, reduced cardiomyocyte apoptosis and myocardium fibrosis, restored myocardial autophagy, and ameliorated left ventricular diastolic dysfunction [213]. Two other animal models showed that trimetazidine improved ventricular diastolic dysfunction and fibrosis in streptozotocin (STZ)-induced diabetic rats by inhibiting ROS production [214,215]. A phase II clinical trial evaluating the efficacy of trimetazidine on left ventricular functions and inflammatory markers in patients with type 2 DM is ongoing (NCT05556005).

### 5.2. Improving Calcium Homeostasis

#### 5.2.1. Istaroxime

Istaroxime is a unique luso-inotropic agent that can increase SERCA2a pump activity and inhibitNa^+^/K^+^ ATPase, thus improving both myocardial relaxation and contraction [216]. In animal studies, istaroxime ameliorated systolic and diastolic dysfunctions [217,218]. Clinical trials demonstrated that istaroxime improved the diastolic index and increased the systolic blood pressure and cardiac index without causing severe adverse events in patients with acute HF [219,220]. A recent study using a type 1 diabetic rat model showed that istaroxime restored SERCA2a activity and reversed streptozotocin-induced diastolic dysfunction and intracellular Ca^2+^ handling [109].

#### 5.2.2. Ranolazine

Ranolazine, a piperazine derivative, is recommended as a second-line anti-anginal agent for patients with chronic stable angina by the guideline of the American Heart Association and the European Society of Cardiology [221,222]. Ranolazine primarily acts by inhibiting the late inward sodium current, which reduces the intracellular Ca^2+^ level due to an enhancement of the Na^+^-Ca^2+^ exchanger by the sodium gradient. As a consequence, ranolazine improves Ca^2+^ handling and ameliorates impaired myocardial relaxation and diastolic dysfunction [223]. Ranolazine also inhibits fatty acid oxidation and improves the efficiency of glucose oxidation [224]. Experimental studies have demonstrated that ranolazine improved cardiac remodeling as well as systolic and diastolic function by restoring Ca^2+^ handling in HF animal models [225]. In a small pilot study on patients with HFpEF, ranolazine decreased left ventricular end-diastolic pressure [226]. In another nonrandomized trial of patients with both systolic and diastolic heart failure, ranolazine treatment improved systolic function and reduced cardiovascular events [227]. In a diabetic rat model, ranolazine improved systolic dysfunction and inhibited myocardial apoptosis by activating the neuregulin pathway [228].

### 5.3. ROS Scavenging

As discussed in the above section, mitochondrial ROS are central in the pathophysiology of diabetic cardiomyopathy. Several ROS scavengers have been evaluated for their possible therapeutic efficacy in diabetic cardiomyopathy. In animal models, vitamin E supplementation has been shown to improve the structural and functional changes and oxidative stress in diabetic hearts [229,230]. However, most clinical trials were disappointing in terms of demonstrating the beneficial effect of reducing the risk of cardiovascular events or preventing and treating HF [231,232,233,234,235], and some even showed harmful results [235,236].

#### 5.3.1. MitoQ

Both the mitochondrial electron transfer chain and NOXs are major sources of ROS in the heart. Scavenging ROS from mitochondria or NOXs may be considered a therapeutic strategy. Increased mitochondrial oxidative stress has been observed in diabetic hearts [237]. MitoQ, a derivative of coenzyme Q, is linked to a lipophilic triphenylphosphonium cation, and this lipophilic conjugated compound can accumulate in mitochondria at high concentrations [238]. *In vitro* studies showed that MitoQ decreased stress-induced mitochondrial ROS and cardiomyocyte hypertrophy [239]. In animal studies, MitoQ treatment improved cardiac apoptosis, fibrosis, hypertrophy, and left ventricular dysfunction in pressure-overloaded hearts [240,241,242]. While these preclinical studies have demonstrated beneficial effects of a mitochondria-targeted antioxidant MitoQ treatment on heart structure and function, clinical trials have not been performed to test its efficacy in patients with heart failure. Moreover, preclinical or clinical investigations are required to test the effect of MitoQ in diabetes-related heart failure.

#### 5.3.2. Coenzyme Q10

Coenzyme Q10 is a component of the mitochondrial electron transport chain that transfers electrons from complexes I to III and functions as a potent ROS scavenger [243]. With the ability to increase ATP production and cellular energy and reduce oxidative damage, coenzyme Q10 has been shown to have cardiovascular protection effects [244]. The level of coenzyme Q10 was found to be decreased in plasma and the myocardium in patients with heart failure, and the plasma concentration of coenzyme Q10 is an independent predictor of survival in HF [245]. A meta-analysis revealed improvement in the left ventricular ejection fraction and the NYHA functional class in patients with HF after the supplementation of coenzyme Q10 [246,247]. A recent RCT that enrolled 420 patients showed beneficial results of long-term supplementation with coenzyme Q10 on cardiovascular mortality, HF hospitalization, and the NYHA functional class [248]. Due to a lack of large-scale RCTs, current studies only support an adjunctive role of coenzyme Q10 in the treatment of HF [243]. Additionally, hyperglycemia-induced oxidative stress plays a major role in diabetic cardiomyopathy. Coenzyme Q10 supplementation attenuated oxidative stress in diabetic mice, and it improved diabetes-induced left ventricular diastolic dysfunction and cardiomyocyte hypertrophy and fibrosis in diabetic mouse models [249,250,251] (Table 3). Further clinical trials with coenzyme Q10 to prevent or treat diabetic cardiomyopathy are required to address this issue.

#### 5.3.3. Elamipretide

Elamipretide (SS-31), another mitochondria-targeted antioxidant, is a small peptide that selectively targets cardiolipin, a phospholipid exclusively found in mitochondria [238]. Cardiolipin increases the peroxidase activity of cytochrome c, which catalyzes substrate oxidation by H_2_O_2_. Elamipretide stabilizes cardiolipin to prevent the cardiolipin-enhanced peroxidase activity of cytochrome c and functions as a mitochondrial ROS scavenger. In animal studies, elamipretide improved ventricular function, while its efficacy for HF in clinical trials showed inconsistent results [264,265,266,267,268]. Further preclinical or clinical investigations are required to evaluate the efficacy of elamipretide in diabetic cardiomyopathy.

#### 5.3.4. GKT137831

The NOX family is another major source of ROS in the myocardium. The inhibition of NOXs with the NOX 1/4 dual inhibitorGKT137831ameliorated diabetic renal complications and some cardiac pathologies in preclinical studies [269,270,271]. Thus, it provides a promising therapy for diabetic cardiomyopathy.

### 5.4. Inhibition of Advanced Glycation End-Products (AGEs)

Chronic hyperglycemia in diabetes increases the formation of AGEs and their receptor, RAGE (receptor for advanced glycation end-products), which contribute to the pathogenesis of diabetic cardiomyopathy [272,273,274]. These AGEs activate RAGE to initiate inflammatory processes [275,276]. Moreover, AGEs crosslink with extracellular proteins such as collagen, myofilaments, and SERCA2a, which increases myocardial fibrosis and impairs myocardial relaxation [69,158,277].

#### 5.4.1. Aminoguanidine

Aminoguanidine is an AGE formation inhibitor that reversed diabetes-induced cardiac remodeling and dysfunction in animal studies [278,279]. However, a clinical trial with aminoguanidine treatment was terminated early due to several side effects [280], and no further clinical trials have been conducted to date to prove its efficacy.

#### 5.4.2. Alagebrium(ALT-711)

Alagebrium (ALT-711), an AGE crosslink breaker, has been studied extensively. In a preclinical study, it attenuated the accumulation of myocardial AGEs and crosslinked collagen. It also decreased the expression of cardiac BNP, a marker of systolic and diastolic cardiac dysfunction, in diabetic rats [252]. In another study with diabetic rats, alagebrium partially improved sarcoplasmic reticulum Ca^2+^ handling and left ventricular diastolic dysfunction [253]. A clinical trial with patients with diastolic HF with or without diabetes revealed that alagebrium reduced the left ventricular mass, improved diastolic dysfunction, and improved quality of life [281]. Later, another trial with patients with systolic HF evaluated the effect of alagebrium on exercise capacity and cardiac function. However, it showed disappointing results [282]. Several clinical trials testing the effects of alagebriumon heart and kidney diseases in patients with or without diabetes were prematurely discontinued due to the termination of financial support [283].

#### 5.4.3. MitoGamide

Methylglyoxal, a byproduct of glycolysis and highly reactive carbonyl species that forms advanced glycation end-products, is elevated in the diabetic myocardium. MitoGamide, a mitochondria-targeted methylglyoxal scavenger, has recently been shown to attenuate left ventricular diastolic dysfunction in type 1 diabetic mice [254]. More preclinical and clinical studies are needed to address its efficacy.

### 5.5. Inhibition of Aldose Reductase

Hyperglycemia in diabetes activates the polyol pathway, one of the pathogenic mechanisms of diabetic cardiomyopathy. Aldose reductase involves the polyol pathway’s first rate-limiting step, which reduces glucose to sorbitol [284,285]. Past studies have established aldose reductase as a critical driver of functional and metabolic impairment in diabetic hearts [286]. Earlier generations of aldose reductase inhibitors were demonstrated to protect against ischemic myocardial injury in animal models [287,288] and to improve cardiac function in patients with type 2 diabetes [289]. However, most of these earlier aldose reductase inhibitors have been unsuccessful in treating diabetic complications due to low binding affinity, specificity, and safety concerns [290].

AT-001is a novel, highly selective, and potent aldose reductase inhibitor with minimal off-target effects [285,291]. AT-001 was well tolerated and safe in the early phases of trials. In a phase IIa trial, treatment with AT-001 for 28 days significantly reduced NT-proBNP levels in patients with diabetic cardiomyopathy [286]. A phase III study (ARISE-HF) is currently evaluating its efficacy and safety in adult patients with diabetic cardiomyopathy at high risk of progression to overt HF (NCT04083339) (Table 4).

### 5.6. Activation of the Neuregulin–ErbB Pathway

The neuregulin family compromises signal proteins that bind to ErbB receptors, a type of EGFR, which are involved in cell growth, differentiation, migration, and survival [130]. Activation of ErbB receptors prevents cardiomyopathy [132,133], while their inhibition causes HF in mice [134,135,136,137]. Neuregulin-1 is mainly synthesized and secreted by endocardial and microvascular endothelial cells in the heart [292]. Experimental studies and clinical trials demonstrated that recombinant human neuregulin-1 reversed ventricular remodeling and improved cardiac function in subjects with HF [293,294,295,296,297]. In preclinical studies on diabetic cardiomyopathy, recombinant human neuregulin-1 improved myocardial function by reducing cardiomyocyte death and cardiac fibrosis in diabetic rats [255,256]. Neuregulin-4 is primarily secreted by brown adipose tissue. Clinical studies revealed that a low serum level of neuregulin-4 was associated with diabetes [298,299]. Emerging evidence has shown that neuregulin-4 improved systolic dysfunction, ameliorated cardiac fibrosis, and prevented cardiomyocyte death in diabetic mice [257].

### 5.7. Activation of Apelin and the Apelin Receptor Pathway

Apelin is a vasoactive peptide that binds to the apelin receptor (APJ), a G-protein-coupled receptor). The apelin/APJ system is present in many tissues and cells and is associated with several physiological functions and pathological conditions. In the cardiovascular system, apelin exerts pleiotropic effects, including positive inotropic activity, reducing cardiac fibrosis, alleviating ventricular hypertrophy, promoting angiogenesis, and relaxing vascular tone [300,301]. Studies demonstrated that apelin-13 treatment improved left ventricular contractility [302,303]. Overexpression of apelin upregulated sirtuin 3, increased myocardial angiogenesis, and ameliorated cardiac hypertrophy and systolic dysfunction in diabetic DB/DB mice [304]. In studies using streptozotocin-induced diabetic mice and kkAy mice, apelin ameliorated diabetic cardiomyopathy by reducing microvascular dysfunction and endothelial function through the endothelial APJ-activated NF-κB pathway [258].

### 5.8. Targeting the Soluble Guanylate Cyclase–cGMP Pathway and Endothelial Dysfunction

Nitric oxide (NO), which is produced by endothelial nitric oxide synthase in the endothelium and the endocardium, spreads to vascular smooth muscle cells or cardiomyocytes to activate the soluble enzyme guanylate cyclase, thus forming cyclic guanosine monophosphate (cGMP). cGMP causes decreases in intracellular free calcium levels, resulting in smooth muscle cell relaxation [120,121]. NO bioavailability is impaired in HF and DM [305].

#### 5.8.1. Cinaciguat

Cinaciguat, a soluble guanylate cyclase activator, exerts beneficial effects on endothelial dysfunction and myocardial remodeling [306,307]. However, in a phase IIb clinical trial with an acute intravenous application, several adverse events, mainly hypotension, caused the study to be prematurely terminated [308]. In a type 1 diabetic rat model, chronic oral administration of cinaciguat prevented myocardial hypertrophy and fibrosis and improved cardiac systolic and diastolic dysfunction in diabetic conditions [259].

#### 5.8.2. Vericiguat

Vericiguatis a soluble guanylate cyclase stimulator that enhances soluble GC activity [309]. Two clinical trials with oral administration of vericiguat showed beneficial results without severe adverse effects in patients with HF with a reduced ejection fraction. In the SOCRATES-REDUCED trial, the high-dose treatment group showed a significant reduction in NT-proBNP levels and a significant increase in the left ventricular ejection fraction [310]. The phase III VICTORIA trial showed that treatment with vericiguat reduced the composite primary endpoint of death from cardiovascular causes or the first HF hospitalization in patients at high risk of heart failure [311]. These results demonstrate its promise as a therapy for diabetic cardiomyopathy, while preclinical and clinical studies are needed to address its efficacy in this disease.

### 5.9. Reducing Toxic Aldehydes

In diabetes, hyperglycemia-associated oxidative stress induces lipid peroxidation of biological membranes to generate reactive aldehydes, such as 4-hydroxy-2-nonenal (4-HNE). These reactive aldehydes crosslink covalently with proteins and DNA to form advanced lipid peroxidation end-products, leading to protein dysfunction, the alteration of intracellular signaling, and subsequent pathological conditions [312,313]. Reactive aldehydes, especially 4-HNE, are related to myocardial remodeling and dysfunction in diabetes [78,314].

Mitochondrial aldehyde dehydrogenase 2 (ALDH2) is the enzyme critical for scavenging toxic aldehydes [284]. Previous studies found decreased expression and activity of ALDH2 in diabetes, which was associated with cardiac dysfunction [80,315]. Overexpression of ALDH2 ameliorates high-glucose-induced cardiotoxicity [316]. The ALDH2 activator, Alda-1, has been shown to enhance ALDH2 activity, attenuate ischemic myocardial injury, and improve high-glucose-induced cardiac fibrosis and necroptosis [79,317]. AD-9308 is a highly selective ALDH2 activator that is more potent than the prototype drug Alda-1. We previously demonstrated that AD-9308 ameliorated diabetic cardiomyopathy through the restoration of ALDH2 activity and by reducing the 4-HNE level [81]. In this study, AD-9308 ameliorated myocardial fibrosis, inflammation, and apoptosis; improved mitochondrial respiration and calcium handling; reduced autophagy in cardiac tissues; and reversed myocardial remodeling and dysfunction in streptozotocin-induced diabetic mice. Our finding regarding AD-9308 shows its promise as a potential therapy for diabetic cardiomyopathy.

### 5.10. Suppression of Inflammation

#### 5.10.1. Phosphoinositide 3-kinaseγ (PI3Kγ) Inhibitor

Phosphoinositide 3-kinaseγ (PI3Kγ) is a lipid and protein kinase expressed in cardiac cells and leukocytes. PI3Kγ plays a critical role in inflammation through leukocyte chemotaxis [318,319]. PI3Kγ has been shown to recruit inflammatory cells and increase fibrosis and maladaptive cardiac remodeling, leading to cardiac dysfunction and a pressure-overloaded heart. Genetic and pharmacological inhibition of PI3Kγ reversed these conditions in mouse models [318,320]. Recently, a pharmacological inhibitor of PI3Kγ, GE21, has been shown to reverse cardiac dysfunction in STZ-induced diabetic mice, which was associated with decreases in inflammation and fibrosis in their hearts [260].

#### 5.10.2. Canakinumab and HMG-CoA Reductase Inhibitors (Statins)

According to the Canakinumab Anti-Inflammatory Thrombosis Outcome Study (CANTOS) trial, canakinumab, a monoclonal antibody that targets IL-1β, provided a strong anti-inflammatory response in high-risk patients with established atherosclerotic disease, leading to a 15% reduction in fatal myocardial infarction, stroke, or cardiovascular death [321]. In another RCT with patients with prior myocardial infarctions and high-sensitivity CRP ≥2 mg/L, canakinumab was related to dose-dependent reductions in HF and HF-related mortality. The highest dose (300 mg) resulted in a 22% reduction in HF [322].

In a following analysis of the CANTOS trial, increasing hsCRP or interleukin-6 (IL-6) was strongly associated with an increased risk of developing diabetes. However, despite large reductions in hsCRP and IL-6, canakinumab did not reduce the incidence of new-onset diabetes during a median follow-up of 3.7 years. Nevertheless, canakinumab reduced HbA1c levels during the initial phase of the study but not in the long-term follow-up [323].

Statins are effective lipid-lowering drugs with a good safety profiles and anti-inflammatory properties. A meta-analysis of six RCTs including more than 110,000 patients with an acute coronary syndrome showed that intensive statin therapy reduced hospitalizations from HF [324]. A combined canakinumab and statin treatment might remove the “residual inflammatory risk” and provide further protection against myocardial infarction and diabetic cardiomyopathy [325,326].

### 5.11. Ameliorating RAAS Axis Activation

#### 5.11.1. Angiotensin 1–7

Angiotensin-converting enzyme 2 (ACE2) cleaves angiotensin II into angiotensin 1–7, which is a negative regulator of the renin–angiotensin system [327,328]. Angiotensin1–7 elicits cardioprotective effects, including vasodilatory, anti-fibrotic, anti-hypertrophic, and anti-thrombotic effects through the activation of *mas* G-protein-coupled receptors in animal models [329]. A loss of ACE2 exacerbates cardiac dysfunction in diabetic hearts [330], whereas enhancing the action of ACE2 attenuates angiotensin-II-induced myocardial hypertrophy, fibrosis, and diastolic dysfunction [331]. In an animal model with *db/db* mice, angiotensin 1–7 administration was shown to improve myocardial hypertrophy, fibrosis, lipotoxicity, and diastolic dysfunction, indicating a beneficial effect of angiotensin 1–7 on diabetic cardiomyopathy [146].

#### 5.11.2. Dipeptidyl Peptidase III

Dipeptidyl peptidase III (DPP III) is an aminopeptidase that cleaves dipeptides from the N-terminals of oligopeptides, but its exact action in the pathophysiology of diseases remains unclear [332]. DPP III has been shown to lower blood pressure in hypertensive mice by digesting the bioactive peptide angiotensin II [333]. Recently, the intravenous administration of recombinant DPP III in type 2 diabetes mice exerted cardioprotective effects, including an amelioration of cardiac diastolic dysfunction and an inhibition of inflammatory cell infiltration and fibrosis in the myocardium, through the cleavage of a cytotoxic peptide that is a fragment of complement component 3 [261].

### 5.12. Inhibition of Fibrosis

#### 5.12.1. Glucose-Dependent Insulinotropic Polypeptide

Glucose-stimulated insulinotropic polypeptide (GIP) is a gut hormone secreted from the intestinal mucosa after nutrient ingestion [334]. The primary physiological role of GIP is to simulate pancreatic β-cells to secrete insulin. The GIP receptor is also expressed in other organs, such as the brain, bones, adipose tissue, and the heart [335,336]. GIP has been shown to inhibit TGF-β expression and ameliorate myocardial hypertrophy and fibrosis in a hypertensive mouse model [336]. GIP also prevented left ventricular hypertrophy and fibrosis in diabetic mice by downregulating the TGF-β pathway [262].

#### 5.12.2. Cathelicidin-Related Antimicrobial Peptide

The endothelial-to-mesenchymal transition has been suggested to be a source of cardiac fibroblasts, which are involved in the pathogenesis of cardiac fibrosis [337]. Hyperglycemia induces the endothelial-to-mesenchymal transition, which leads to increases in collagen deposition and ventricular diastolic dysfunction in diabetic mice [338]. Cathelicidin-related antimicrobial peptide (CRAMP) is a naturally occurring peptide that was initially identified as an antimicrobial peptide [339,340]. In an animal model, it was shown to ameliorate cardiac dysfunction and adverse cardiac remodeling following myocardial infarction by enhancing the engraftment of bone marrow cells in the myocardium [341]. CRAMP attenuated the endothelial-to-mesenchymal transition and fibrosis in diabetic mouse hearts by inhibiting TGFβ/Smad signaling [263].

## 6. Conclusions

This review summarizes the emerging therapies for diabetic cardiomyopathy targeting specific molecular mechanisms. Among these potential drugs, AT-001 and trimetazidine are the most promising ones and are currently being tested in RCTs for diabetic cardiomyopathy. In addition, preclinical studies have supported the therapeutic effects of neuregulin-1 and vericiguat for diabetic cardiomyopathy, which have been effective for treating HF in clinical trials. However, they have not been specifically tested in clinical trials for diabetic cardiomyopathy. Moreover, preclinical studies support the therapeutic efficacy of AD-9308, angiotensin (1-7), and PI3Kγ inhibitors such as GE21 for diabetic cardiomyopathy, but evidence from clinical trials is lacking.

## Figures and Tables

**Table 1:** Stages of diabetic cardiomyopathy.

Stages	Stage 1 (Early)	Stage 2 (Middle)	Stage 3 (Late)	Stage 4 (Late)
Morphological features	Normal LV wall thickness, size, and mass	Slightly increased LV wall thickness, size, and mass	Significantly increased LV wall thickness, size, and mass	Significantly increased LV wall thickness, size, and mass
Functional changes	Normal or slightly diastolic dysfunction	Diastolic dysfunction with normal or slightly systolic dysfunction	Diastolic and systolic dysfunction	Diastolic and systolic dysfunction
Cellular and metabolic abnormalities	Insulin resistance with reduced glucose uptake Increased circulating free fatty acids and fatty acid utilization Ca^2+^ homeostasis impairment	AGE formation Cardiomyocyte death Myocardial fibrosis Increased oxidative stress and inflammation Reduced nitric oxide Activated RAAS Mild CAN	Extensive myocardial fibrosis Microvascular changes Severe CAN	Extensive myocardial fibrosis Microvascular changes Severe CAN CAD
Echocardiographic methods for diagnosis	Sensitive echocardiographic techniques such as speckle-tracking echocardiography and tissue Doppler imaging	Conventional echocardiography with sensitive echocardiographic techniques	Conventional echocardiography	Conventional echocardiography

AGE, advanced glycated end-products; CAD, coronary artery disease; CAN, cardiac autonomic neuropathy; RAAS, renin–angiotensin II–aldosterone system.

**Table 2:** Novel biomarkers for diagnosing diabetic cardiomyopathy.

Promising Biomarkers		References
sST2	An interleukin-33 (IL-33) decoy receptor that ameliorates the Th2 inflammatory response. Increased plasma levels of sST2 have been linked to a worse prognosis of HF.	[179,180,181]
Galectin-3	A lectin family protein that has been associated with fibrosis and inflammation in heart failure. It is elevated in diabetic patients with a mild depressed ejection fraction and is associated with a diminished global longitudinal strain. It is an easy and reproducible tool for the evaluation and follow-up of diabetic cardiomyopathy. It was approved by the FDA as a novel biomarker for predicting adverse cardiovascular events.	[182,183,184,185]
lncRNAs	Long noncoding RNAs (lncRNAs)are a diverse subgroup of noncoding RNAs that are epigenetic regulators of gene expression. Circulating lncRNA serum levels are associated with cardiac remodeling and are positively correlated with cardiac diastolic dysfunction.	[7,186,187]
GDF-15	Expressed in response to oxidative stress and inflammation. GDF-15 levels predict diabetic cardiomyopathy.	[188,189]
TGF-β	A fibrogenic cytokine that promotes extracellular matrix accumulation. Higher plasma levels of TGF-β are associated with both DM and diastolic dysfunction.	[190,191,192]
IGFBP7	Regulates the IGF signaling pathway and is related to insulin resistance. IGFBP7 is correlated with DM with diastolic dysfunction.	[192,193]
**Other candidate biomarkers**		
Activin A	A protein produced by epicardial adipose tissue. It is correlated with both myocardial glucose metabolism abnormality and left ventricular remodeling.	[194,195]
H-FABP	It has an independent association with outcomes in patients with HF. It enables the detection of early myocardial injuries in patients with metabolic syndrome or DM.	[196,197,198]
TNF-α	The level was increased in diabetic patients with diastolic dysfunction. It might be used for the early detection of diabetic cardiomyopathy.	[192,199]
ICTP and PIIINP	Circulating collagen turnover biomarkers are associated with adverse cardiac outcomes and can be used to predict HF progression.	[200,201]
Cardiotrophin-1	A glycoprotein 130 and a potent inducer of myocardial hypertrophy. It is correlated with blood glucose and ventricular hypertrophy. It is less specific for dilated cardiomyopathy.	[202,203]

GDF-15, growth differentiation factor-15; H-FABP, heart-type fatty acid binding protein; ICTP, collagen type I carboxy-terminal telopeptide; IGFBP7, insulin-like growth factor binding protein 7; lncRNAs, long noncoding RNAs; PIIINP, procollagen type III N-terminal propeptide; sST2, soluble form of suppression of tumorigenicity 2; TGF-β, transforming growth factor-β; TNF-α, tumor necrosis factor-α.

**Table 3:** Effective treatment of diabetic cardiomyopathy in preclinical studies.

Drug	Diabetic Animal Model	Mechanism	Effects	Reference
Trimetazidine	High-fat diet and streptozotocin induced type 2 diabetes in rats	3-ketoacyl coenzyme A thiolase inhibitor (inhibition of β-oxidation of fatty acid)	Ameliorated metabolic disturbance and insulin resistance, ↓LVEDP, ↓cardiomyocyte apoptosis, ↓myocardial fibrosis, restored cardiac autophagy	[213]
Trimetazidine	Streptozotocin-induced diabetes in rats	3-ketoacyl coenzyme A thiolase inhibitor	↓cardiac hypertrophy and fibrosis, ↑dP/dtmax, ↓LVEDP, ↓inflammation and oxidative stress, ↓Nox2 and TRPC3	[214]
Trimetazidine	Streptozotocin-induced diabetes in rats	3-ketoacyl coenzyme A thiolase inhibitor	↓malondialdehyde level, ↓LVEDP, ↓myocardial fibrosis, ↓ROS production	[215]
Istaroxime	Streptozotocin-induced diabetes in rats	Increases SERCA2a pump activity and inhibits Na+/K+ ATPase	Improved LV diastolic dysfunction, ↑SERCA2 protein level and activity, improved intracellular Ca^2+^ handling anomalies	[109]
Ranolazine	Streptozotocin-induced diabetes in rats	Inhibits the late inward sodium current	↓cardiac hypertrophy, ↑LVEF, ↓cardiomyocyte apoptosis, ↑NOTCH1/NRG1 signaling pathway	[228]
Coenzyme Q10	Streptozotocin-induced diabetes in mice	Mitochondrial ROS scavenger	↓oxidative stress, improved LV diastolic dysfunction, ↓cardiomyocyte hypertrophy and fibrosis, ↓connective tissue growth factor and β-myosin heavy chain	[249]
Coenzyme Q10	*db/db* mice	Mitochondrial ROS scavenger	↓oxidative stress, improved LV diastolic dysfunction, ↓cardiomyocyte hypertrophy and fibrosis	[250]
Coenzyme Q10	Streptozotocin-induced diabetes in mice	Mitochondrial ROS scavenger	↓oxidative stress, improved LV diastolic dysfunction, improved LV remodeling (↓cardiomyocyte hypertrophy, cardiac fibrosis, and apoptosis), ↓proinflammatory mediators	[251]
Alagebrium	Streptozotocin-induced diabetes in rats	AGE crosslink breaker	Restored LV collagen solubility and ↓crosslinked collagen, ↓cardiac BNP, ↓cardiac AGE levels and AGE receptor, ↓connective tissue growth factor and collagen III expression	[252]
Alagebrium	Streptozotocin-induced diabetes in rats	AGE crosslink breaker	Partially alleviated diastolic dysfunction, ↑SERCA2a and RyR2 protein content, improved SR Ca^2+^ reuptake	[253]
MitoGamide	Akita mice	Mitochondria-targeted methylglyoxal scavenger (preventing AGE formation)	Improved LV diastolic dysfunction	[254]
Recombinant human neuregulin-1	Streptozotocin-induced diabetes in rats	Signaling molecule binding to epidermal growth factor (ErbB) receptor	↑dP/dtmax, ↓LVEDP, ↓cardiomyocyte apoptosis, ↓cardiac fibrosis, ↓collagen type I and III	[255]
Recombinant human neuregulin-1	Streptozotocin-induced diabetes in rats	Signaling molecule binding to epidermal growth factor (ErbB) receptor	↑dP/dtmax, ↑myocardial angiogenesis, ↑expression of vascular endothelial growth factor and angiopoietin-1	[256]
Neuregulin-4	Streptozotocin-induced diabetes in mice	Signaling molecule binding to epidermal growth factor (ErbB) receptor	↓cardiac hypertrophy, ↑LVEF, ↓cardiac fibrosis and apoptosis, activated autophagy via the AMPK/mTOR signaling pathway	[257]
Apelin	Streptozotocin-induced diabetes in kkAy mice	Binding to APJ receptor	↑LVEF, ↓cardiac hypertrophy and fibrosis, ↑myocardial angiogenesis, ↓microvascular dysfunction, ↓expression of adhesion molecules	[258]
Cinaciguat	Streptozotocin-induced diabetes in rats	Soluble guanylate cyclase activator	↑plasma and myocardial cGMP;↓the expression of PDE-5;↑PKG activity;↑systolic function (preload recruitable stroke work) and diastolic function (tau);↓myocardial hypertrophy, apoptosis, and fibrosis;↓oxidative stress	[259]
AD-9308	Streptozotocin-induced diabetes in mice	Mitochondrial aldehyde dehydrogenase 2 activator	↓serum 4-HNE levels and 4-HNE protein adducts in cardiac tissue; improved LV diastolic and systolic function;↓myocardial fibrosis, inflammation, and apoptosis; improved mitochondrial function, SR Ca^2+^ handling, and autophagy regulation	[81]
GE21	Streptozotocin-induced diabetes in mice	Inhibitor of PI3Kγ	Improved systolic and diastolic dysfunction on echocardiography, ↓cardiac inflammation and fibrosis	[260]
Angiotensin 1–7	*db/db* mice	Negative regulators of the renin–angiotensin system	Improved diastolic dysfunction, ↓myocardial hypertrophy and fibrosis, ↓lipotoxicity, ↓adipose inflammation, ↓myocardial oxidative stress, ↑phosphorylation of myocardial Erk1/2, ↓expression of myocardial protein kinase Cα and PKCβ1, ↑adipose triacylglycerol lipase	[146]
Dipeptidyl peptidase III	*db/db* mice	Cleavage of a cytotoxic peptide that is a fragment of complement component 3	Improved diastolic dysfunction, ↓cardiac inflammation and fibrosis	[261]
GIP	*db/db* mice	Suppression of TGF-β2	↓cardiac hypertrophy and fibrosis, ↓TGF-β pathway, ↓myosin heavy chain β and TGF-β2	[262]
CRAMP	Streptozotcin induced diabetes in mice	Inhibits endothelial-to-mesenchymal transition	Improved systolic and diastolic dysfunction, ↓cardiac fibrosis, ↓endothelial-to-mesenchymal transition, ↓TGF-β/Smad signaling, ↓AMPKa1 signaling	[263]

AGE, advanced glycation end-product; AMPK, adenosine 5‘-monophosphate-activated protein kinase; BNP, B-type natriuretic peptide; cGMP, cyclic guanosine monophosphate; CRAMP, cathelicidin-related antimicrobial peptide; Erk1/2, extracellular signal-regulated kinase 1/2; GIP, glucose-dependent insulinotropic polypeptide; 4-HNE, 4-hydroxy-2-nonenal; LV, left ventricular; LVEDP, left ventricular end-diastolic pressure; LVEF, left ventricular ejection fraction; mTOR, mammalian target of rapamycin;NOTCH1, notch homolog 1; Nox2, NADPH oxidase 2; NRG1, neuregulin 1; PDE-5, phosphodiesterase-5; PI3Kγ, phosphoinositide 3-kinase γ; PKC, protein kinase C; PKG, cGMP-dependent protein kinase; RyR2, ryanodine receptor 2; SERCA2a, SR Ca^2+^ pump; SR, sarcoplasmic reticulum; TGF, transforming growth factor; TRPC3, transient receptor potential channel 3.

**Table 4:** Ongoing clinical trials for the treatment of diabetic cardiomyopathy.

Drug	Mechanism	Phase of Development	ClinicalTrials.gov Identifier
AT-001	Aldose reductase inhibitor	Phase III	NCT04083339
Trimetazidine	3-ketoacyl coenzyme A thiolase inhibitor	Phase I	NCT05556005
Dapagliflozin	Sodium–glucose transporter-2 inhibitor	Phase IV	NCT04200586
Dapagliflozin	Sodium–glucose transporter-2 inhibitor	Not applicable	NCT04591639
Dapagliflozin	Sodium–glucose transporter-2 inhibitor	Phase II	NCT04304560
Eplerenone	Mineralocorticoid receptor antagonist	Phase I	NCT01794091

## Data Availability

Not applicable.
